# Refining a Video and Text Message Intervention (STAR, Skills Training in Active Recovery) to Prevent the Onset or Escalation of Posttraumatic Stress and Opioid Misuse Among Recent Sexual Assault Survivors: Community Engaged Study

**DOI:** 10.2196/72095

**Published:** 2025-08-18

**Authors:** Kate Walsh, Anne Marie Schipani-McLaughlin, Cynthia Stappenbeck, Shahzarin Khan, Anna Woodworth, Sanika Panwalkar, Ronald Acierno, Amanda K Gilmore

**Affiliations:** 1 Department of Psychology, University of Wisconsin–Madison, 1202 W Johnson Street, Madison, WI, 53706, United States, 1 6082628992; 2 Department of Health Policy and Behavioral Sciences, School of Public Health, Georgia State University, Atlanta, GA, United States; 3 Department of Psychology, Georgia State University, Atlanta, GA, United States; 4 Department of Applied Health Science, School of Public Health–Bloomington, Indiana University, Bloomington, IN, United States; 5 Anthropology Division, SUNY Polytechnic Institute, Utica, NY, United States; 6 Department of Psychiatry and Behavioral Sciences, McGovern Medical School, UTHealth Houston, Houston, TX, United States

**Keywords:** intervention development, community advisory board, sexual assault, early intervention, text message, survivor

## Abstract

**Background:**

Sexual violence is prevalent, and the consequences can be chronic and impairing. However, few interventions exist to prevent the onset or escalation of posttraumatic stress symptoms and opioid misuse among recent sexual violence survivors.

**Objective:**

This study describes a collaborative process of updating an integrated postsexual assault video and developing an SMS text messaging intervention program with a community advisory board (CAB) of sexual assault survivors.

**Methods:**

Research team members met virtually for six 60‐90-minute meetings with a 5-member CAB of sexual assault survivors with diverse racial and gender identities located throughout the United States. CAB members provided feedback on written documents detailing an adapted video script and newly developed text intervention to address the risk of substance misuse and posttraumatic stress disorder symptoms following sexual assault. CAB members also received SMS text messages to provide feedback from the end-user perspective.

**Results:**

We identified overarching themes to improve relatability (destigmatize and increase awareness of support, reduce technical language, and increase representation in actors), content (increase social support, include substance-related assault, and suggest activities), and wording (normalize different terms for sexual assault and reduce insensitive language) for the video intervention. For the text intervention, we identified themes relating to acceptability (timing, frequency, and format of texts), relatability (having an avatar introduce the program and identifying the study name in messages), content (messaging), and wording (increasing clarity)*.*

**Conclusions:**

Findings reinforce the importance of including community members’ perspectives and suggestions to improve the acceptability and relatability of interventions, including the video and SMS text message intervention described here.

## Introduction

Sexual assault (SA) is a significant public health problem that affects more than 1 in 2 women, nearly one-third of men, and 2 in 5 trans people in the United States [1,2]. SA survivors experience numerous deleterious consequences, ranging from physical health conditions such as headaches, chronic pain, and irritable bowel syndrome [3], to mental health conditions including posttraumatic stress disorder (PTSD), depression, suicidality, and substance misuse and disorders [4]. Medical, mental health, lost work productivity, criminal legal, and property damage costs associated with SA amount to more than US $3.1 trillion (2014 US dollars) over survivors’ lifetimes in the United States [5].

PTSD and substance misuse are particularly pernicious and frequently co-occurring difficulties following SA [6-8]. Indeed, meta-analyses suggest that 75% of SA survivors meet criteria for PTSD 1 month after assault and 40% meet criteria 12 months after assault [9]. Opioid misuse correlates strongly with both SA and PTSD [10,11]. Among women seeking a sexual assault medical forensic exam (SAMFE), 21% reported nonmedical use of prescription opioids in the year prior to the assault, and 15.6% reported using them nonmedically in the 6 weeks after the assault [12]. However, few interventions target PTSD and opioid misuse, and none are tailored for the immediate post-SA period.

Early interventions that are perceived positively by SA survivors are key to preventing chronic and impairing symptoms [13]. The Prevention of Post-Rape Stress (PPRS) video is a 9-minute intervention that has been used in 3 randomized controlled trials (RCTs) with SA survivors presenting for a SAMFE [14-17]. The original PPRS video was developed by clinician-researcher experts in SA interventions that drew on three frameworks relevant to trauma, substance misuse, and depression recovery: (1) emotional processing theory [18], which posits that fear responses to trauma-related stimuli can be extinguished through exposure; (2) substance use relapse prevention approaches [19] that encourage avoiding substance misuse activators and instead focusing on coping in ways that are incompatible with substance use; and (3) behavioral activation approaches [20] that encourage engaging in healthy coping activities like spending time with supportive people and taking walks. Therapeutic exposure, relapse prevention, and behavioral activation have strong evidence for efficacy in reducing PTSD [21], substance misuse [22], and depression [23,24], respectively. The PPRS includes a single narrator describing possible reactions to SA, including posttraumatic stress symptoms, substance misuse, and depression, along with ways to address these reactions by recognizing and preventing trauma-related avoidance (ie, therapeutic exposure), recognizing substance misuse activators, and engaging in activities incompatible with substance use such as spending time with people who do not use substances or engaging in activities to fight depression like exercising.

In 2 RCTs, the PPRS was associated with reductions in PTSD and depression for survivors with a prior history of SA [15,25] and reductions in cannabis use among those with preassault use [16,17] when compared to treatment as usual (TAU). TAU consists of the standard provision of a SAMFE that can include injury treatment, pregnancy and sexually transmitted infection prophylaxis, evidence collection, or connection to other referrals and resources. The PPRS video has been associated with reductions in cigarette smoking [26], alcohol use [17], and suicidality [27] compared to TAU. The latter 2 findings have been for survivors with preassault alcohol use and those with heightened distress at the SAMFE.

In a third RCT, a mindfulness video was delivered as an active control condition in comparison to the PPRS and TAU. Compared to TAU, the mindfulness video was effective at reducing prescription opioid misuse 1.5 months post-SA for those with a prior SA history; the PPRS video did not differ significantly from TAU [12]. This finding fits with broader literature suggesting that mindfulness interventions are effective at reducing opioid misuse and chronic pain [28-30]. Mindfulness interventions also have been used to effectively address PTSD and depression among SA survivors [31,32] and have been found acceptable among women with comorbid PTSD and substance use disorder more generally [33]. Therefore, to target PTSD, depression, cannabis use, cigarette use, alcohol use, suicidality, and opioid misuse, it is essential to incorporate mindfulness components into the PPRS video.

Despite the positive effects of PPRS, our finding that the video is more effective for survivors with a previous history of SA [12,15,25] suggests that it may be important to increase relevance to survivors broadly in future iterations of the video. Models of intervention development emphasize the importance of adapting interventions prior to rigorous effectiveness testing [34], and formative work like eliciting feedback from a community advisory board (CAB) can be an important initial step. CABs often consist of community members who have lived experience and expertise relevant to the topic in question. In this case, CAB members with SA and SAMFE experiences could provide feedback that increases both the relevance and acceptability of the video. Indeed, CABs have been shown to improve the quality and relevance of research for the communities the research impacts [35]. CABs have successfully informed interventions for specific populations in prior work [36,37] and thus could enhance the relevance of the intervention for SA survivors in this study.

Another challenge was that, understandably, some survivors were distressed at the time of the SAMFE [38], which happens in the acute postassault time frame, and may not have absorbed the information presented in PPRS as readily. Thus, providing the same core intervention content in the days and weeks following the SAMFE may help improve retention and subsequent outcomes for these individuals. An SMS text messaging program delivered after the SAMFE may be a viable strategy to push this content from the video over a longer period of time and via a different medium. A November 2024 report from the Pew Research Center found that 98% of Americans own a cellphone and 91% own a smartphone [39], so an SMS text messaging program could be viable. Indeed, SMS text messaging programs have been used to successfully improve mental health and substance use symptoms [40]. Indeed, a brief intervention for PTSD and alcohol misuse among SA survivors found that delivering 4 brief SMS text messages was highly acceptable to survivors and associated with reductions in PTSD symptoms, weekly drinks, and heavy episodic drinking at 8-week follow-up [41]. However, to our knowledge, an SMS text message program to address comorbid PTSD and other substance use has not been evaluated, particularly as a follow-on intervention amplification method after the acute timeframe-based intervention.

This study describes a formative collaborative process of working with a CAB of SA survivors to refine content for a new video called Skills Training in Active Recovery (STAR). STAR integrates the principles of the PPRS and mindfulness videos to better address PTSD, depression, and substance misuse including opioid misuse. To make STAR and TextSTAR more relevant and acceptable to women and nonbinary SA survivors, the CAB provided feedback on ways to make the interventions more relatable, accessible, understandable, and helpful. We analyzed feedback from meetings through thematic analysis and discussions with the broader research team and made decisions collectively about how to refine the interventions to be responsive to this feedback. This was an important formative step before evaluating the acceptability and preliminary effectiveness of STAR and TextSTAR in a pilot RCT with recent SA survivors presenting for a SAMFE.

## Methods

### Recruitment of CAB Members

The target population and study protocol were considered when recruiting and selecting CAB members. Because SA disproportionately impacts women and those who identify outside the gender binary [1,2], those who present to SAMFE programs for care are disproportionately women [42], and our prior trials have only included women [16,17], we focused efforts on refining the content of this video and SMS text messaging program with women and nonbinary people specifically. Additionally, because people of color in the United States experience SA at high rates [1] and are often excluded from research, it was important to ensure that CAB members were racially diverse people drawn from across the United States. Given the focus on opioid and other substance misuse, we were interested in including those with substance misuse experiences. Additionally, because participants in the broader research study would be recruited at a SAMFE, we sought CAB members who had some experience with receiving post-SA medical care.

The study team developed a flyer that we distributed via email to 11 organizations across 10 states. The flyer stated that we were seeking SA survivors to provide feedback on an intervention for recent SA survivors. Those who were interested could scan the QR code or click the study link in the advertisement to complete a short screening survey that included questions about demographics, experiences of unwanted or nonconsensual sexual touching or oral, anal, or vaginal penetrations, medical care and services received after the unwanted or nonconsensual experience, substance use, and interest or willingness to be on a CAB focused on improving an intervention. We used these responses to select CAB members who were women or nonbinary, racially and ethnically diverse, and had SA, SAMFE, and varied substance misuse experiences, including opioid misuse.

### CAB Participant Characteristics

CAB members were 5 women or nonbinary survivors of SA located in 4 states across the United States. Our budget for the project was limited, so we opted to recruit a smaller number of CAB members to participate regularly in a larger number of meetings. We wanted CAB members to have the time to share feedback and be heard, which the smaller group size accomplished. The smaller CAB allowed for more engagement with the material and more meaningful opportunities for CAB members to provide feedback and discuss suggestions with each other. CAB members were all 18 years or older and ranged in age from 25 to 54 years. Members had diverse racial identities, including White, South Asian, African American, and American Indian.

### Ethical Considerations

The proposed CAB process to refine these interventions was considered “not human subjects research” (IRB 2023‐1219).

### CAB Process

CAB meetings were held in a HIPAA (Health Insurance Portability and Accountability Act)-compliant, passcode-enabled Zoom room. Meetings were 1 to 1.5 hours long and were audio-recorded and transcribed by an author (SP). Because we were not asking CAB members to share personal experiences with each other but rather to share feedback on the video and SMS text messaging program, we opted to audio-record meetings to ensure that we accurately recorded specific and detailed feedback. Recruitment emails and flyers clearly stated to potential CAB members that they would not be asked to share details of their nonconsensual sexual experiences with others and that we would be audio-recording meetings, so those who did not feel comfortable could make an informed decision not to participate. To ensure safety and comfort for all CAB members, the screening form confirmed that CAB members would be willing to have their cameras on and ensure that they were in a private space during meetings. We also developed a protocol that we reviewed with CAB members individually and in the first meeting about how to handle others unexpectedly entering their space or needing to leave during a CAB meeting. Although we set the expectation that CAB members did not need to share trauma details, the first author (KW), who led the meetings, is a licensed clinical psychologist and offered to speak with any CAB members after meetings if they found any of the content or discussions challenging or needed referrals for mental health resources.

**Figure 1.**
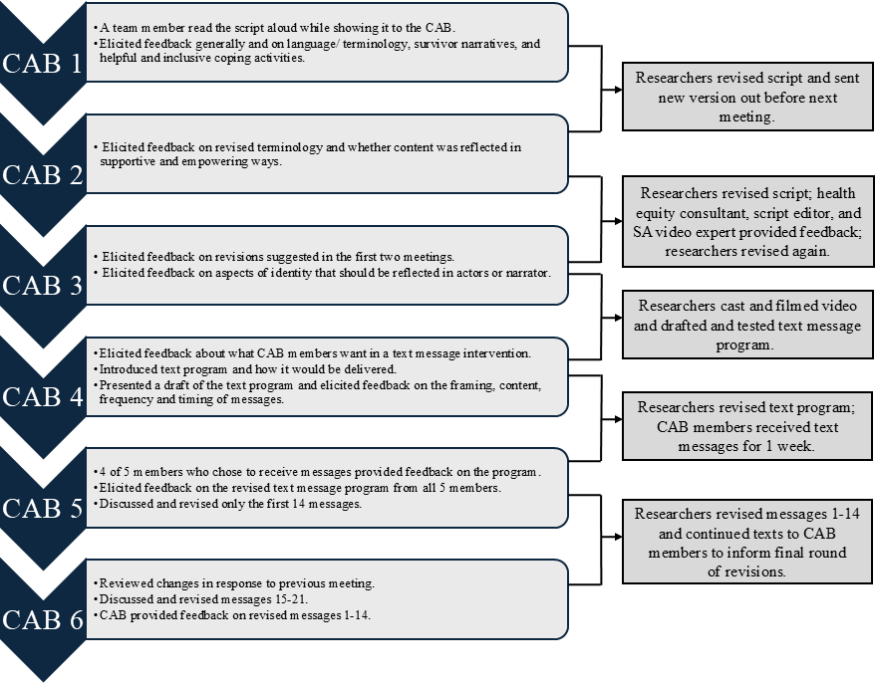
CAB meeting tasks and activities between meetings. CAB: community advisory board.

Prior to each meeting, CAB members typically received slides or other content (eg, a video script, a document with SMS text messaging program messages) to familiarize themselves with the content we would be discussing. Two members of the research team (KW and SP) met with the CAB on 3 occasions to elicit feedback on the video script and on 3 occasions to elicit feedback on the texting program (Figure 1). CAB members were compensated US $50/hour for time spent in meetings, and they received an optional US $100 bonus if they agreed to receive the study text messages and provide feedback from the end-user perspective.

### Iterative Video Intervention Refinement Process

The 5-member research team began refining the original video script to incorporate (1) SA survivor narratives to educate and normalize common experiences, (2) coping skills and tips told from survivor perspectives and modeled on screen in some cases (eg, how to do gradual exposure), (3) mindfulness skills (eg, urge surfing) to address opioid misuse specifically, and (4) distress tolerance skills (eg, temperature, intense exercise, and paced breathing) [43]. We also developed an empowering ending to the video that involved the narrator, actors, and many extras with diverse backgrounds and identities to highlight that anyone can experience SA and there is a large community of support.

In the first meeting, a team member read the script aloud while showing it to the CAB and asked for general feedback as well as feedback on language or terminology, examples of things that should be represented in survivor narratives, and activities survivors could suggest that would feel both helpful and inclusive. In between meetings, the research team revised the video script based on CAB feedback and sent these revised versions out to the CAB, so they could have a few days to review the changes before the next meeting. In the second meeting, we revisited the script to obtain additional feedback on some of the changes we made to ensure that revised terminology felt appropriate and that content was reflected in supportive and empowering ways. In the third meeting, we revisited themes from the first 2 meetings to ensure additional edits appropriately reflected earlier suggestions. We also asked the CAB for feedback to inform casting decisions prior to filming the video. Specifically, CAB members were asked to describe who they envisioned in each of the roles and what aspects of identity were important to have included or represented in the video.

### Additional Consultation

Based on CAB feedback, we also had a health equity consultant review and edit the video script to ensure that it was inclusive for neurodiverse SA survivors. A scriptwriter then provided edits to make the video more conversational. Next, an SA expert consultant who developed the original video script reviewed and provided edits on the final version.

### SMS Texting Program Refinement Process

The study team constructed approximately 3 weeks of SMS text messages using content and phrasing from the video. This material was organized into supportive, educational, and skills-focused messages, and we evaluated a version that had content types grouped by week and a version that had content types woven throughout. In the fourth meeting, we introduced the SMS text messaging intervention and how team members planned to implement it. We first elicited general feedback about what CAB members would like to see from an SMS text messaging intervention. We then presented a draft of the SMS text messages the research team had created and asked for feedback on the framing of the program, content type, message ordering, and frequency and timing of messages.

In between meetings, the research team received SMS text content to gain clarity on how it felt to receive messages and refine the program with internal feedback. In the first CAB meeting, we described how the research team envisioned the texting program would be delivered and what alternatives had been considered to provide context. Specifically, although an interactive texting program staffed by those with SA advocacy expertise could be incredibly helpful and tailored to the specific concerns survivors experienced in a specific moment, we did not have the personnel or funding available to meet this need. Additionally, we had considered sending short video links (edited from the longer video) via texting. However, based on research showing the value of brief texting for SA survivors with PTSD and alcohol misuse [41], we opted to keep written texts limited largely to 160 characters or less (ie, the character limit for a single SMS text message). In the CAB meeting 4, which was the first that focused on feedback on the texting program, we asked CAB members for (1) general feedback on the structure of the proposed program, (2) feedback on the frequency and timing of messages, and (3) what would make the CAB likely to open or read the messages and try some of the skills. We also showed CAB members a few sample messages and elicited feedback. Prior to the fifth meeting, we invited CAB members to receive a week’s worth of SMS text messages so we could get feedback about the end-user perspective of receiving the messages, including the order in which the first week of messages was delivered. Then, in the fifth meeting, 4 out of 5 members who chose to receive messages started by providing feedback on the program. One CAB member had a family emergency and opted not to receive SMS text messages during these weeks. A team member then presented the revised 21-day SMS text messaging program and elicited content-related feedback from all 5 members. In the allotted meeting time, we only discussed and revised the first 14 messages. Prior to the sixth meeting, the research team revised messages 1‐14 based on CAB feedback and continued sending messages to CAB members to inform the final round of revisions. We asked for feedback on how it felt to receive the messages, the timing of when to receive messages during the day, whether any messages did not sit well with participants, and whether any messages should be edited. We also went through the first 2 weeks of SMS text messages and addressed wording, content, and ordering changes. For the third SMS text message meeting, CAB members received an additional week of SMS text messages and weighed in on the content, wording, and ordering of the third week of messages.

### Reflexive Thematic Analysis

Using the 6 steps of reflexive thematic analysis [44,45], the first author (KW), who led the CAB meetings, read and reread transcripts to familiarize herself (step 1) with the data and took notes both during CAB meetings and while reading. Next, she generated initial codes (step 2) and searched for themes (step 3). With the research team, she reviewed themes (step 4) and defined, named, and organized themes according to intervention content (video vs text; step 5). These codes and themes were iteratively discussed with the broader research team, and an initial draft of the results was drafted, shared with CAB members, and iteratively refined (step 6). Additionally, because the purpose of the meetings was to improve the interventions, we have described the study team’s response to the suggested changes.

### Reflexivity Statement

Braun and Clarke [46] eschew a one-size-fits-all checklist for qualitative research and instead emphasize *reflexive openness* in their values-based guidelines for qualitative research. Predominantly, the women research team is composed of scholars who largely have doctoral-level training in psychology and public health. Several team members are licensed clinical psychologists who have provided trauma-focused and substance misuse–focused therapy for SA survivors and who strive to include the communities they serve in the design and production of research. Team members also have extensive experience collaborating with SAMFE programs to conduct research to better support SA survivors in the immediate aftermath of SA. Collaborating with the CAB illuminated biases in how the research team viewed content and messaging that could, in practice, be less acceptable or even off-putting for survivors. We practiced openness to receiving feedback and being responsive to concerns raised throughout the intervention refinement process by engaging in open discussion with the shared understanding that the research team and CAB both wanted to produce the most helpful possible content.

## Results

### Video Intervention Suggestions

Suggestions for video intervention improvements can best be described using a funnel or upside-down triangle where at the broadest level, CAB members made suggestions to improve the *relatability* of the video; at the middle level, they made suggestions to improve the *content* of the video, and at the bottom level, their suggestions focused on improving the *wording* of the video. Specific subthemes within each domain are identified in the text and Table 1.

**Table 1.** Summary of community advisory board recommendations and research modifications for the video and text messaging interventions*.*

Domain	Challenge	CAB^a^ suggestion	Researcher modification
Video challenge			
Relatability	Technical language	Do not use “mindfulness”	Described techniques but did not label “mindfulness”
	Representation in actors	Seek actors with visible physical disability	Asked casting company for extra with visible disability
Content	Include messaging about social support	Explicit suggestions to seek support from one’s community	Created more dialogue between the actors telling their stories in the video to model support
	Include substance-related assault	Substance-related assault notably missing	Had actor describe a substance-involved experience and feelings of self-blame
Wording	Normalize use of various SA^b^ terms	Use the word “violated” in addition to “rape/sexual assault/trauma” Include discussion from the narrator and actors about different ways to talk about what happened “Harm” too vague	Narrator expressly says there are many different ways to talk about what happened and actors use different terms when describing their own experiences including “rape, sexual assault, and violated”
	Reduce insensitive language	Use “challenge yourself to” instead of “do this” to recognize difficulty and reduce ableism	Replaced recommendations to “do this” with “challenge yourself to”
Text program challenge			
Acceptability	Responding to messages	Do not require responses to keep “low pressure”	Removed response requirements
	Frequency	No more than once/day	Created a daily text program
	Timing	Might be burdensome to anticipate when they’ll need support Transition from late afternoon to early evening	Sent around 4 pm each day but ask for feedback about timing
	Formatting	Prefer opening question followed by tip or suggestion preferred	Use mostly opening questions followed by tip or suggestion preferred
Relatability	Introducing the program and texts	Have avatar introduce program so there is a “person” sending texts Start texts with “STAR^c^ study:”	Crafted introductory text from “Shari from the STAR study” with photo Began each text with “STAR Study:”
Content	Messaging	Change the content of the educational text	Removed language about symptoms passing with time and included validating statement
	Ordering	Place general text before specific	Reordered so general text came before specific
Wording	Clarity	Clarify tempo of breathing Expand on verb “use”	Added the word “slowly” before counting instructions during breathing exercise Added words “drugs or alcohol”
	Specificity	“Approach” does not have teeth “Danger” is not specific	Changed to “confront” Changed to “violation or traumatic event”

a CAB: community advisory board.

b SA: sexual assault.

c STAR: Skills Training in Active Recovery.

#### Relatability

Suggestions to improve the relatability of the video centered on ways to make it more appealing and survivor-centered. Examples included specific suggestions to destigmatize SA experiences and increase awareness of support, reduce technical language to make the video more appealing and easier for an average person to understand, and ensure diversity and representation in actors and in the kinds of experiences and narratives reflected in the video.

##### Destigmatize Assault and Increase Awareness of Support

A CAB member suggested adding a statement to the beginning of the video, destigmatizing the assault and highlighting the community of support:

*I think in the beginning like a plain language statement of like, you’re not alone. Like there is a community. What happened to you is real, and you should feel empowered to share your story because the more that we don’t talk about this, the more it happens in silence to other people. Not to say it’s our responsibility to stop abusers, but like, it’s only a positive feedback loop. By us harboring the guilt and not talking about it, basically.*

To address this suggestion, we have the survivor-actors say, “What happened to you was not ok, it was not your fault, and you are not alone” at the beginning of the video.

##### Reduce Technical Language

The CAB noted that some of the content from the original video was technical and potentially difficult to follow for individuals in acute distress. For example, CAB members recommended not using the term “mindfulness” and instead focusing on the techniques:

*I think there’s a lot of mention of “mindfulness. I don’t know, maybe it’s just like a generational thing, where it’s a lot of focus right now for a lot of things. Where right now if somebody tells to me, oh, am I having an anxiety attack? Why don’t you try mindfulness? It just feels a bit less. But the techniques that was said was very specific. So it’s just about rephrasing, you know, if somebody comes to me and say this is something bad that has happened to me. Why didn’t you try this technique instead of mentioning mindfulness?*

The research team removed the term “mindfulness” from the script and instead described the actual techniques.

##### Increase Diversity and Representation in Actors

One goal for the new video was to move away from a single narrator describing intervention content to a format that allowed the narrator and multiple survivor-actors to personalize the information presented and show a breadth of experiences and coping strategies. The research team planned to show a narrator and actors who varied in terms of race and gender expression to make the content feel relatable to a larger swath of the population. However, the CAB expanded on this intention by recommending that we include actors of all shapes and sizes and at least one actor with a physical disability. These conversations allowed the research team to write casting calls to yield a diverse cast, seek an extra with a visible physical disability, and prioritize representation in the selection of actors.

### Content

At the middle level, CAB members made suggestions to enhance or include new content that they viewed as highly relevant to the healing process. Content suggestions included promoting efforts to seek social support early and often, describing substance-facilitated SA, and providing feedback on suggested activities to cope, distract from substance use, or increase positive feelings.

#### Increase Social Support Content

CAB members noted that none of the skills the video promoted could be effectively implemented without social support. For example, one CAB member said:

*Not too much of what was said or suggested is gonna more than likely happen without support first. And I only heard support in like the middle part of it as being a big, you know, or mentioned, it wasn’t like support was like the number one because first of all, without support, with no support or...when you get a medical attention, if they don’t believe you, you’re more than likely to turn to those drugs and feelings. Yeah, but so the...the part that I felt was missing was the stressing support as number one. And, like, one of the first priorities.*

In response to suggestions to increase social support content, we had the narrator and actors emphasize the importance of social support early and throughout the video. Specifically, we created more dialogue between the actors telling their stories in the video and responding empathically to others who described what happened to them to model what support could and should look like. We also explicitly encourage seeking out supportive community members throughout the video and show clips of survivors calling supportive people and meeting up with them for a walk or other activity. We also end the video by encouraging survivors to consider other supports, including talking to a professional, if they find that their symptoms are not improving over the next few weeks.

#### Include Substance-Involved SA Content

CAB members raised that substance-involved assaults were especially difficult to identify as assault, and shame and guilt were common immediate reactions. They believed it was critical to include some acknowledgment of these kinds of experiences within the survivor narratives to be clear that this is a type of assault:

*I was surprised to not see anything in it about or for survivors who were under the influence during their assault. I thought that might be applicable. I think it would be helpful, but I don’t have like a clear thought of the messaging around it yet.*

Based on this suggestion, the study team revised the script to include a narrative from an actor-survivor that included feeling a lot of shame and self-blame for experiencing assault while using substances.

#### Activity Suggestions

One specific question the research team had for CAB members was which coping activities we should highlight and which may be less useful to emphasize. Organized religion (eg, going to church) was identified as an activity to exclude from the examples we provided:

*It suggests like things you can do to help you when you’re feeling like inundated or overwhelmed by feelings and it mentions prayer and going to church. While that is definitely helpful for some people, that’s probably something that is better to stay away from considering this is a general audience. Like you don’t know what the person is bringing, right, because a lot of people in that moment have given up on religion and so I think it’s really insensitive to suggest that because, like, what, you pray for what?...those particular words really take away from the authenticity of what is trying to be accomplished here. So I guess trying to stay away so much from religion, sorry, structurally religious suggestions. Spiritually, I guess, reflection, meditation I think, like, that’s totally different than prayer and going to church because I also think it’s, you know, specific to Christianity.*

### Wording

At the narrowest level, CAB members made wording suggestions about terminology to use to describe experiences of SA and to minimize potentially insensitive language.

#### Normalize Use of Various SA Terms

CAB members all used different terms to describe their SA experiences. They encouraged us to include acknowledgment of different terms to normalize the language survivors may use for SA experiences:

*There were a lot of, um, like emotions and tactics that I know during my first assault I would not have like understood, like my emotions were so out of whack and I wouldn’t have been able to make sense of a lot of it. Which I think might be why it resonated more with people who, um, had an earlier experience. I think kind of going back and focusing on the feelings of it rather than the term, I don’t know, I’m not married to this term that I’m about to give, but like ‘being violated.’ I think that was a feeling that resonated with me and was clear even though I didn’t understand it at the time. So maybe going back to the basics with some stuff like that.*

Another CAB member said:

*I think telling survivors that they can talk about what happened without labeling it, without saying this is that or it felt like that, and just identifying that your feelings are valid and you can feel them. You don’t have to do anything, you don’t have to make any decisions. You can still talk to somebody about what happened without saying what it was, cause I think a lot of the time people don’t wanna talk about it because they’re not sure if it was assault, or if it was worth talking to anybody about and it’s like, you can just talk about that without knowing. That’s completely real and valid and shared by so many people.*

The research team addressed this concern by including the offered terms in narrative examples from the survivor-actors and using these terms interchangeably throughout the video.

#### Reduce Potentially Insensitive Language

CAB members noted some places where the video unintentionally came off as insensitive or ableist. For example, one CAB member said:

*There is some language at the end about like make yourself get out of bed and like don’t blame yourself that also rely on that like there’s a standard way of doing things, that there is a standard level of capability. Like, saying, like, you know, make yourself do these things, that’s a very neurotypical piece of advice and later, when I thought about like how to rephrase that I liked “challenge yourself to like go beyond your, your, your like bubble of comfort*.”

Another CAB member noted:

*When I was reading don’t blame yourself at the end, umm, I was thinking one thing that to me feels more powerful is saying that this is not your fault. Umm, I think that that might feel a little bit more reassuring. And I think that that particularly applies to the substance-use, umm, scenarios.*

In response to these comments and suggestions, we changed our language to “challenge yourself” and “it’s not your fault.” Similarly, there were suggestions to change older, colloquial terms that have come to have a negative or stigmatizing connotation to less stigmatizing terminology. For example, a CAB member said:

*I also want to note that there was a part early that said, like, we want to acknowledge that what you are feeling is real, you’re not going crazy, and I also think that’s a bit ableist because that’s very loaded language too. I think that could be something more like, umm, you know, this is not all in your head.*

To address this language concern, we used the CAB member’s language of “it’s not all in your head.”

### SMS Texting Intervention Suggestions

#### Acceptability

##### Responding to Messages

The budget for the pilot would not allow for real-time monitoring and responding to SMS texts, which is clarified to participants in the informed consent as well as the introductory text of the SMS text messaging intervention. However, the team initially considered whether to assess message engagement by encouraging participants to respond to messages, including by typing back answers to questions or just rating the content of the message using “thumbs up” or “thumbs down” emojis. Although the CAB liked the “thumbs up” and “thumbs down” option, they preferred not requiring any sort of response because it was “low pressure.” To ensure that no pressure was placed on participants in the SMS text messaging program, we ultimately opted to ask about the helpfulness of the SMS text messaging program in weekly surveys instead of after each message.

##### Timing

In an ideal world, survivors would be able to choose when to receive messages, but not all people know when they might need more support throughout the day, so we asked CAB members to weigh in on the preferred time of day for SMS text message delivery. While some CAB members had no preference for time of day, a few noted that late afternoon, when transitioning from the day into the evening, where plans might be less structured, could be a helpful time to receive messages encouraging them to seek support, although this assumes that people have more structured days and less structured evenings. We tested the late afternoon time-out with CAB members when they received SMS text messages; although one indicated that receiving messages in the morning might help her get out of bed on tough days, nearly everyone else reported liking the late afternoon time. Thus, we plan to initially pilot the SMS text messaging program being delivered in the late afternoon.

##### Frequency

Prior to receiving the SMS text messages, CAB members noted that they would not like to have messages more than once per day, and they were not sure that they would even want daily SMS text messages. After receiving daily SMS text messages for 2 weeks, we reassessed, and they all appreciated the daily SMS texts and thought once per day was the right frequency.

##### Format of Messages

CAB members viewed differently structured sample messages and strongly preferred messages that opened with a question and then provided a tip or suggestion (eg, Feeling down? Challenge yourself to get out of the house and spend time with supportive people even when you don’t feel like it).

### Relatability

#### Introducing Text Messages

The CAB recommended that we consider creating an avatar or identity that survivors would connect to the texting program to increase the acceptability and likelihood of engaging with the texting content. To address this suggestion, we had the narrator of the video film a brief clip introducing the texting program, and we sent an introductory message from “Shari from the STAR Project” with a photo of the narrator to accompany the SMS texts. CAB members preferred the introductory SMS text message from Shari as opposed to clicking a video link to view the introduction. CAB members were agnostic about including a photo, so we excluded it. Members of the research team proposed having participants save the STAR study phone number as a contact, and CAB members suggested having the messages open with “STAR:” to orient them to the content. We included all of these recommendations in the version of the text program we formally tested.

### Content

#### Messaging

One CAB member experienced additional violence just before receiving the test SMS text messages and reported that the messages were particularly helpful and validating. Another said:

*I especially liked the ones where they, where you gave specific advice, like steps to do. I thought that was a nice change of pace for a lot of automated messages. It didn’t feel like in the void*.

However, CAB members also had suggestions for improvement in content. For example, one of the educational SMS texts was intended to provide information about the fight, flight, or freeze response but differed from other texts in that there was not a corresponding tip or action suggested. The research team tried out various “tips” that included just letting natural reactions happen, but the CAB suggested that a message that was simply about validating those experiences was sufficient. Based on this feedback, we decided not to include a tip and instead added validation.

#### Ordering

CAB members also recommended switching the ordering of SMS text messages so a general text about avoidance came before a more specific text about how to address avoidance. The study team also received SMS text messages on multiple occasions and recommended including more supportive SMS text messages early in the program with more skill and activity suggestion text messages later in the program.

### Wording

In the research team’s efforts to make messages brief, we occasionally risked being unclear or lacked adequate specificity to be helpful.

#### Clarity

The CAB identified places where certain messages lacked clarity or could be misinterpreted. For example, one SMS text message read, “Trouble sleeping? Close your eyes and breathe in while you count to 4, hold your breath while you count to 4, then release your breath while you count to 4.” A CAB member noted:

*You haven’t given any point of reference for how fast or slow you’re supposed to be counting, so theoretically that could actually make it worse if you’re counting way faster than you’re supposed to be or in the alternative it could actually make someone hold their breath*.

We revised the message to include the word “slowly” before “breathe.” Another SMS text message initially read, “Want to use? Some strategies people use to just wait out urges include taking long, slow deep breaths in and out, or picturing a peaceful scene.” One CAB member felt that the verb “use” was specific to a certain type of drug use and recommended that we clarify the question to include “drugs or alcohol.” We made this change to improve clarity and relevance.

#### Specificity

The CAB also identified messages that lacked adequate specificity. For example, one SMS text message read, “Approaching scary or difficult things can be hard,” and a CAB member noted that the word approach “had no teeth” and a word like “confront” would be more aligned with what we were trying to convey.

## Discussion

### Summary

This paper describes a researcher-CAB collaboration to develop a 15-minute video and 21-day SMS texting program to prevent the onset and escalation of PTSD symptoms and opioid misuse for recent SA survivors who received a SAMFE. A prior 9-minute video was associated with reductions in a variety of mental health and substance use difficulties [17,25]. However, the revised video described here was designed to resonate more with recent survivors by including actors who described different kinds of experiences and different coping strategies in addition to a narrator, more relaxation and mindfulness skills to address opioid misuse (eg, urge surfing), and more empowering content. Consistent with other community-engaged approaches to developing interventions [36,37], CAB members provided feedback to improve the relatability, content, and wording of the video and SMS text messaging program to ensure greater acceptability and relevance to survivors with various backgrounds and experiences. The resulting interventions expand on a growing body of early interventions for recent SA survivors that typically target PTSD [13,47] and address comorbid substance use and PTSD symptoms [41]. Early interventions like the video and SMS text messaging program can be relatively easily administered and low cost, which has important implications for broad dissemination [47].

Some of the most critical suggestions the CAB made were around including more empowering statements and language to reduce the stigma of experiencing SA that often leads people to avoid talking about what happened [48], forego needed health care [49], and even experience revictimization [50]. CAB suggestions to prioritize seeking social support early and throughout the video and SMS text messaging program also fit with a large literature suggesting that social support is critical to recovery among sexual violence survivors [51-54]. However, some survivors may require more support than a video or SMS text messaging program can offer in developing a network of supportive people. Considering additional ways to enhance social support will be crucial for future work.

### Limitations and Future Research

Although CAB members were diverse across a variety of dimensions, including gender, SA and substance misuse histories, and geographic locations, they comprised a small group of survivors at different stages of healing. Data on acceptability from a separate sample of survivors with more recent SA experiences will be critical to establish the utility of the interventions, and a fully powered RCT will be necessary to establish the efficacy of the interventions. Additionally, the CAB and the intervention refinement process only focused on women and nonbinary people, in part, because these groups are substantially more likely than men to seek SAMFE care [42]. Men also experience SA [1] but may experience shame and disempowerment that are entangled with feelings of emasculation due to rape myths that SA victimization does not happen to men [55]. Instead of trying to develop a one-size-fits-all intervention that risks not feeling personalized or relatable to anyone, a CAB of men that is led by a man could elicit feedback about how supportive content might be framed so that male survivors find it relatable and acceptable. Additional work will be needed to develop a video and SMS text messaging intervention appropriate for men. This study also did not include non–English-speaking individuals or those from non-US contexts. CABs should be developed for these populations to refine the messaging in ways that feel tailored to these groups to achieve maximum benefit.

Although SA tends to be concentrated among adolescents and young adults [1] who are likely to be more familiar with technology, the development of a texting program and reliance on Zoom to hold CAB meetings to connect with survivors from across the country may mean we are hearing from and creating interventions for those who are more technologically savvy. Two of our CAB members who stated they were not technologically savvy regularly joined Zoom from their smartphones and had highly positive feedback on the texting program. Additionally, in our pilot trial, we have budgeted for the small percentage of survivors who lack a phone to receive a prepaid smartphone and service for the duration of the study. Nonetheless, future efforts should be made to ensure equitable access to both the intervention development or refinement process and the interventions.

### Conclusions

This study highlights the importance of collaborating with people with lived experiences of violence to develop relevant and acceptable early interventions to prevent the onset and escalation of mental health and substance use difficulties.

## Abbreviations

CAB: community advisory board
HIPAA: Health Insurance Portability and Accountability Act
PPRS: Prevention of Post-Rape Stress
PTSD: posttraumatic stress disorder
RCT: randomized controlled trial
SA: sexual assault
SAMFE: sexual assault medical forensic exam
STAR: Skills Training in Active Recovery
TAU: treatment as usual
